# Learning from nature: HIV elite controllers as blueprints for a functional cure

**DOI:** 10.1186/s12985-025-03042-9

**Published:** 2025-12-24

**Authors:** V. Kalidasan, Kumitaa Theva Das

**Affiliations:** 1 Institute for Research in Molecular Medicine, Universiti Sains Malaysia, Pulau Pinang, Malaysia; 2 Department of Microbiology, Faculty of Medicine, Manipal University College Malaysia, Melaka, Malaysia

**Keywords:** HIV/AIDS, Elite controllers, Functional cure, Natural immunity, Viral reservoirs

## Abstract

Antiretroviral therapy (ART) has transformed HIV infection into a manageable chronic condition but remains non-curative and requires lifelong adherence. Rare cases of long-term remission following stem cell transplantation (SCT) have demonstrated the possibility of viral eradication, yet this approach is not scalable or safe for global implementation. A unique subset of people living with HIV, known as elite controllers (ECs), can naturally suppress replication-competent HIV without ART, often for decades, while maintaining stable CD4 + T cell counts and showing no signs of disease progression. Their ability to sustain treatment-free viral suppression provides compelling evidence that durable remission is biologically achievable, offering a model for cure research. This review synthesizes current evidence on the biology of ECs, encompassing viral, intrinsic antiviral, genetic, and immune mechanisms that underlie natural control. It also examines demographic and clinical characteristics, landmark case reports, and the broader public health implications of elite control. Finally, insights from EC biology are discussed in relation to translational strategies such as gene editing, immune modulation, therapeutic vaccination, and reservoir-targeting approaches designed to mimic or reinforce natural control mechanisms. Understanding the determinants of viral suppression in ECs provides a biological blueprint for the development of a functional cure. As the field advances toward scalable, safe, and durable remission strategies, lessons from ECs remain central to achieving long-term viral control and ultimately ending the HIV epidemic.

## Background

HIV/AIDS has shaped global health for more than four decades, leaving a legacy of loss, activism, and resilience [1, 2]. The introduction of antiretroviral therapy (ART) transformed HIV infection from a universally fatal disease into a manageable chronic condition [3–6]. According to UNAIDS, an estimated 40.8 million people were living with HIV in 2024, including 39.4 million adults and 1.4 million children, with 1.3 million new infections and 630,000 AIDS-related deaths reported that year [7]. While ART has prevented millions of deaths and enabled long-term survival, it is not curative. While the global decline in new infections reflects advances in treatment and prevention, ART has clear limitations. Lifelong adherence is required, as treatment interruption results in rapid viral rebound. Long-term use is associated with toxicities, immune dysregulation, and the persistence of latent reservoirs. These underscore the need for strategies that can achieve durable, treatment-free remission.

Recognizing these challenges, the International AIDS Society’s Global Scientific Strategy for an HIV Cure (2021) emphasized research priorities focusing on reservoir biology, mechanisms of natural control, and the development of gene- and immune-based therapies [8]. Among these priorities, individuals who naturally suppress HIV without ART provide the most compelling evidence that sustained remission is biologically possible. These rare cohorts demonstrate that the human immune system, under certain genetic and immunological configurations, can achieve equilibrium with a replication-competent virus [9]. Examples of natural resistance to infection in humans strengthen this concept. Individuals exposed to *Mycobacterium tuberculosis* who fail to develop latent infection exhibit “early clearance,” reflecting innate immune resistance [10]. Likewise, carriers of the sickle-cell mutation are protected from severe malaria [11]. Similar biological resilience has been described in HIV. The Pumwani sex worker cohort in Nairobi revealed women who remained uninfected for decades despite repeated exposure, associated with protective human leukocyte antigen (HLA) alleles [12, 13]. Other resistant individuals carry the CCR5Δ32 mutation or mount HIV-specific immune responses despite exposure [14–16]. Together, these findings demonstrate how host genetics and immune factors can influence susceptibility to infection and the course of disease.

Among those who do acquire HIV, clinical outcomes vary widely. Most experience progressive immune decline, whereas a minority, termed long-term non-progressors (LTNPs), maintain low viral loads and stable CD4 + T cell counts for years without therapy [17–19]. Within this group, an exceptionally rare subset, elite controllers (ECs), achieve durable suppression of plasma viremia to undetectable levels without ART. Understanding how ECs maintain durable remission provides the conceptual framework for developing immune- and gene-based interventions that can induce similar outcomes in the broader HIV-positive population. As such, ECs exemplify the biological feasibility of a functional cure and serve as a cornerstone for modern HIV cure research.

## HIV cure strategies: from sterilizing approaches to functional remission

Efforts to cure HIV infection have evolved along two conceptual pathways: sterilizing cures and functional cures [20, 21]. A sterilizing cure aims to eradicate all replication-competent HIV from the body, achieving plasma viral loads below the limit of detection even in the most sensitive assays (< 1 copy/mL). In contrast, a functional cure resembles cancer remission, in which people living with HIV (PLHIV) maintain long-term virological control and immune health in the absence of therapy, despite the persistence of low-level latent virus (< 50 copies/mL). A hybrid approach seeks to combine both strategies, reducing the size and activity of the viral reservoir while reinforcing immune-mediated containment. The ultimate goal across all cure paradigms is to establish an intervention that is safe, scalable, and globally accessible, enabling sustained remission and preventing transmission [22].

In recent years, several landmark cases have demonstrated that durable ART-free remission is achievable under specific circumstances. The first of these, the Berlin patient (Timothy Ray Brown) [23], achieved sustained remission following allogeneic stem cell transplantation (SCT) from a donor homozygous for the CCR5Δ32 mutation [24–26]. Similar outcomes were later observed in the London patient (Adam Castillejo) [27], City of Hope patient (Paul Edmonds) [28], Düsseldorf patient (Marc Franke) [29], New York patient [30], Geneva patient [31], the second Berlin patient [32], French patient [33], Chicago patient [34], and the Oslo patient [35]. In nearly all cases, viral remission followed transplantation from CCR5Δ32/Δ32 donors, rendering new CD4 + T cells resistant to HIV infection by eliminating the principal co-receptor for viral entry [36].

These clinical successes have provided critical proof-of-concept that HIV eradication or long-term remission is biologically possible. However, the SCT-based strategy remains unfeasible for widespread implementation. The CCR5Δ32 mutation is rare and geographically restricted, found at higher frequencies in Northern European populations but nearly absent in Sub-Saharan Africa, Asia, and Latin America [37, 38]. Moreover, SCT carries substantial procedural risks such as graft-versus-host disease (GVHD), opportunistic infection, and treatment-related mortality, and is ethically justified only for patients requiring transplantation for hematologic malignancies [39]. Although PLHIV face an increased risk of Hodgkin and non-Hodgkin lymphoma compared to HIV-negative individuals, these cancers occur in diverse contexts, influenced by age, CD4 + T cell counts, viral co-infections, chronic inflammation, and ART regimens [40]. Consequently, SCT represents a therapeutic exception rather than a generalizable cure approach. These cases, while extraordinary, have reshaped HIV cure research by proving that durable remission can be achieved through targeted genetic modification of the host’s immune system [41]. They have catalysed new lines of investigation into cell- and gene-based therapies that could replicate the protective effects of CCR5Δ32 homozygosity without the need for transplantation. Current efforts may include gene editing to disrupt CCR5 or excise integrated provirus [42–45], as well as chimeric antigen receptor (CAR) therapies [46, 47] designed to enhance antiviral immunity, and stem cell reprogramming to confer resistance or enhance immune clearance. These strategies aim to achieve long-term remission safely, without the morbidity of SCT or the constraints of donor matching.

## Elite controllers: determinants and biological diversity in natural HIV control

Interest in non-progressors (NPs) has deepened as a window into the natural dynamics of HIV–host interaction. Among PLHIV, approximately 5 to15% maintain CD4 + T cell counts above 500 cells/µL for at least five years, and sometimes a decade or longer, without ART [48–50]. These individuals, referred to as long-term non-progressors (LTNPs), remain clinically stable with low or moderate viral loads. Within this broader group lies an even smaller subset known as the elite controllers (ECs) or “elite suppressors,” who sustain plasma viral loads below the detection limits of standard assays (< 50–75 copies/mL) without therapy [51]. While LTNPs are defined primarily by preserved immune function, ECs are distinguished by their capacity for near-complete virological suppression in the absence of treatment [52].

**Fig. 1:**
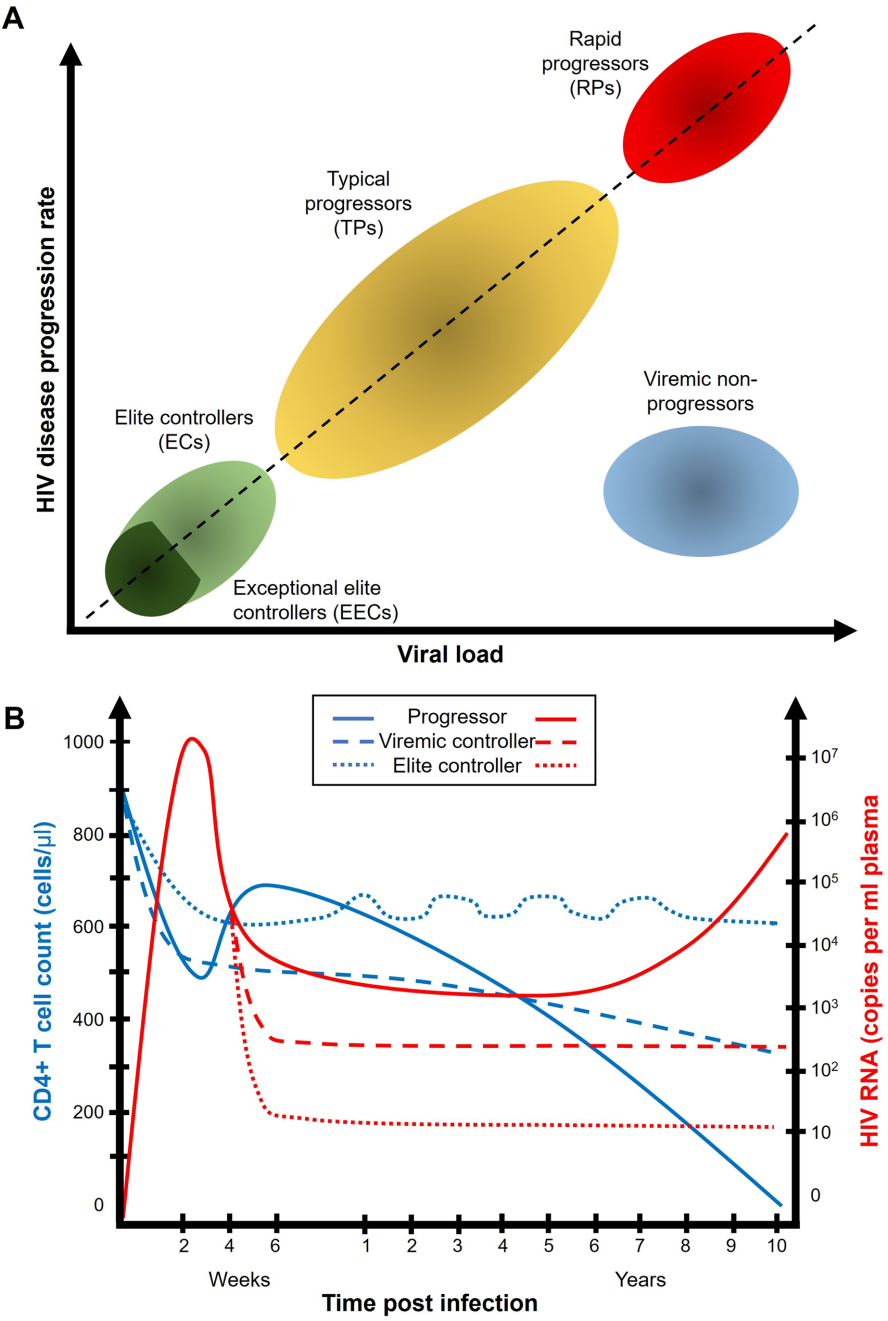
Classification and longitudinal dynamics of HIV disease progression phenotypes. (**A**) Schematic representation of major HIV progression categories based on plasma viral load and rate of disease progression. EECs and elite controllers ECs occupy the lowest end of the spectrum, maintaining minimal viremia and slowest progression, while TPs and rapid progressors RPs exhibit increasing viral loads and accelerated immune decline. Viraemic non-progressors sustain moderate viremia with stable CD4 + T cell counts. (**B**) Representative trajectories of CD4 + T cell counts (blue) and plasma HIV-1 RNA levels (red) over time following infection in progressors, viraemic controllers, and ECs. Progressors show rapid immune depletion and viral rebound; viraemic controllers maintain intermediate suppression; ECs preserve long-term immune stability with persistently undetectable viremia

ECs are exceedingly rare, representing less than 1% of all PLHIV [53, 54]. They are found across genders, ethnicities, and routes of transmission, although certain genetic backgrounds appear to be overrepresented in certain populations. Protective HLA alleles, particularly HLA-B*57, HLA-B*27, and HLA-B*58, and CCR5Δ32 heterozygosity are more frequent among ECs, suggesting that immune presentation efficiency and viral entry restriction play pivotal roles in spontaneous control. Subtype B infection predominates in EC cohorts, possibly reflecting the historical concentration of studied populations rather than subtype-specific effects. A small fraction of ECs, termed exceptional elite controllers (EECs), demonstrate even more profound control, maintaining undetectable viremia (< 20–50 copies/mL) for more than 25 years with little or no recoverable replication-competent virus [19, 55]. These individuals illustrate the extreme end of the HIV control spectrum and provide critical mechanistic clues for achieving durable remission. Table 1 summarizes the defining features of typical progressors, LTNPs, ECs, and EECs, while Fig. 1 illustrates the corresponding trajectories of viral load and CD4 + T cell preservation.

**Table 1:** Definitions and key characteristics of HIV disease progression phenotypes

Phenotype	Viral load	CD4 + T cell count	Clinical course	ART requirement	Representative features
Typical Progressors	> 10⁴–10⁵ copies/mL	Progressive decline (< 200 cells/µL within 5–10 years)	Develop AIDS-defining illnesses without ART	Require continuous ART	High viral replication, immune activation, reservoir expansion
Rapid Progressors	> 10⁵ copies/mL	Rapid decline (< 200 cells/µL within 2–3 years)	Early AIDS onset, poor immune recovery	Immediate ART required	Weak HIV-specific immune responses, high inflammation
Long-Term Non-Progressors (LTNPs)	< 10⁴ copies/mL	Stable > 500 cells/µL for ≥ 5 years	Clinically asymptomatic without ART	Not required short-term	Low-level viremia, preserved CD4⁺ T cell function
Viremic Controllers	50–2,000 copies/mL	Stable or slowly declining	Slow disease progression without ART	May not require ART initially	Partial viral control, intermediate immune activation
Elite Controllers (ECs)	< 50 copies/mL (below clinical assay limit)	Stable > 500 cells/µL for ≥ 10 years	No clinical progression without ART	Not required	Durable suppression of replication-competent virus; polyfunctional CD8⁺ T cells and NK cells
Exceptional Elite Controllers (EECs)	< 20–50 copies/mL (undetectable by ultrasensitive assays)	Stable for ≥ 25 years	No clinical or immunologic progression	Not required	Sterilizing or near-sterilizing control; defective provirus; protective HLA alleles (e.g., HLA-B*57*, *B*27)

Demographically, ECs appear to be more frequent among women than among men. Cohorts from South Africa, Brazil, Thailand, and Europe have reported female predominance, with women comprising up to 91% of ECs in certain studies [56–59]. A Brazilian study similarly found 61% female representation among ECs [60]. Women generally exhibit lower plasma HIV-1 RNA levels than men, even in early infection, often accompanied by higher CD4 + T cell counts [61–64]. These observations are partly explained by the reduced surface expression of the CCR5 co-receptor on female CD4 + T cells, which limits viral entry and replication [65]. However, despite lower viremia, disease progression at comparable viral loads tends to be faster in women, potentially due to higher baseline immune activation and inflammation.

Gender-based immunological and genetic differences contribute further to this divergence [66–68].Many immune-related genes are located on the X chromosome, and although one X chromosome is typically inactivated, several immune loci escape silencing [69]. Among these are toll-like receptors (TLRs), including TLR-7 and TLR-8, which detect single-stranded HIV RNA and initiate type I interferon signalling [70, 71]. These X-linked advantages enhance innate immune responsiveness; plasmacytoid dendritic cells from women produce significantly higher levels of interferon-α following stimulation with HIV-1 TLR7 ligands compared with those from men, leading to stronger downstream CD8 + T cell activation [72, 73]. This enhanced early sensing likely contributes to the higher frequency of elite control observed in women, but it also promotes chronic immune activation, which may explain the gender-related differences in inflammation-driven comorbidities.

Hormonal factors further shape HIV susceptibility and disease course. Oestrogen influences mucosal integrity, immune modulation, and the vaginal microbiome [74]. Experimental data from rhesus macaques demonstrate the protective effects of oestrogen against simian immunodeficiency virus (SIV), whereas in humans, the effect appears to be concentration- and phase-dependent [75]. High oestrogen levels correlate with reduced HIV acquisition, partly through down-regulation of CCR5 and dampened inflammatory signalling [71]. Oestrogen can also directly repress HIV transcription by recruiting oestrogen receptors to the viral long terminal repeat (LTR) [76]. Conversely, fluctuations of oestradiol and progesterone during the menstrual cycle influence replication dynamics, with increased HIV expression during the mid-proliferative phase and decreased replication in the mid-secretory phase [77].

Collectively, these observations suggest that elite control emerges from a convergence of genetic, immunologic, and hormonal factors that together favour effective viral containment. In women, reduced CCR5 expression, X-linked immune gene activity, and oestrogen-mediated antiviral effects may contribute to superior early immune recognition and control. However, these same features may predispose to higher immune activation and inflammatory sequelae, potentially offsetting some of the protective advantages. Understanding how biological sex, genetic architecture, and immune modulation intersect to influence elite control offers valuable insight into the heterogeneity of natural remission. Moreover, these findings emphasize the necessity for HIV cure strategies that accommodate biological diversity, ensuring that interventions designed to reproduce EC-like states are equally effective across genders, genetic backgrounds, and viral subtypes.

## Elite and exceptional HIV controller: clinical evidence and mechanistic insights

ECs and EECs have provided compelling evidence that long-term remission of HIV is biologically achievable without ART. These individuals sustain undetectable viral loads, preserve normal CD4 + T cell counts, and show minimal disease progression despite harbouring replication-competent virus. Over the past three decades, an increasing number of reports have characterized this rare phenomenon, highlighting the diversity of mechanisms that can lead to viral containment.

Large international studies, including the International HIV Controllers Study [78], the French ANRS CO21 CODEX cohort [79], and long-term analyses from the Spanish Exceptional Elite Controller (EEC) cohort [19], have collectively defined the epidemiological and immunogenetic spectrum of elite control. These cohorts confirm that fewer than 1% of PLHIV achieve spontaneous suppression of plasma viremia, with consistent enrichment of protective alleles such as HLA-B*57:01, HLA-B*27:05, and HLA-B*81:01. The French CODEX cohort also demonstrated that approximately 30% of ECs eventually lose virological control [80], while the Spanish EEC study identified a subset maintaining suppression for more than 25 years, often with >90% defective proviral genomes and no detectable replication-competent virus [81]. These findings highlight that elite control exists on a continuum, from durable functional remission to near-sterilizing cure, reflecting a dynamic equilibrium between host and virus rather than a fixed clinical state.

The earliest well-characterized ECs were described in the United States in 2012 [82]. These individuals exhibited undetectable plasma viremia by single-copy assays (< 1 copy/mL) and carried at least one protective HLA-B*57 allele, conferring highly effective cytotoxic T lymphocyte (CTL) responses. Despite the presence of a replication-competent virus, recovery was difficult, indicating strong post-entry restriction. This finding established a paradigm: that efficient antigen presentation and robust cellular immunity can achieve durable, ART-free suppression. Similar insights emerged from diverse populations, confirming that elite control is not geographically or demographically confined, but rather arises from converging host-virus interactions.

Multiple case reports and cohort analyses have since refined this understanding. African ECs, such as two women who maintained undetectable viral loads for over seven years, demonstrated distinct pathways to control: one through vigorous HIV-specific CD8 + T cell responses, the other through pre-existing HIV-specific CD4 + T cell immunity [83]. The Sydney Blood Bank Cohort (SBBC) presented a unique natural experiment, where an individual infected with *nef*/*3’LTR-*deleted HIV-1 exhibited near-sterilizing control for nearly four decades [84]. In this case, viral attenuation, combined with a protective HLA-B*57 genotype and strong cellular responses, created a synergy between viral weakness and host strength.

**Fig. 2:**
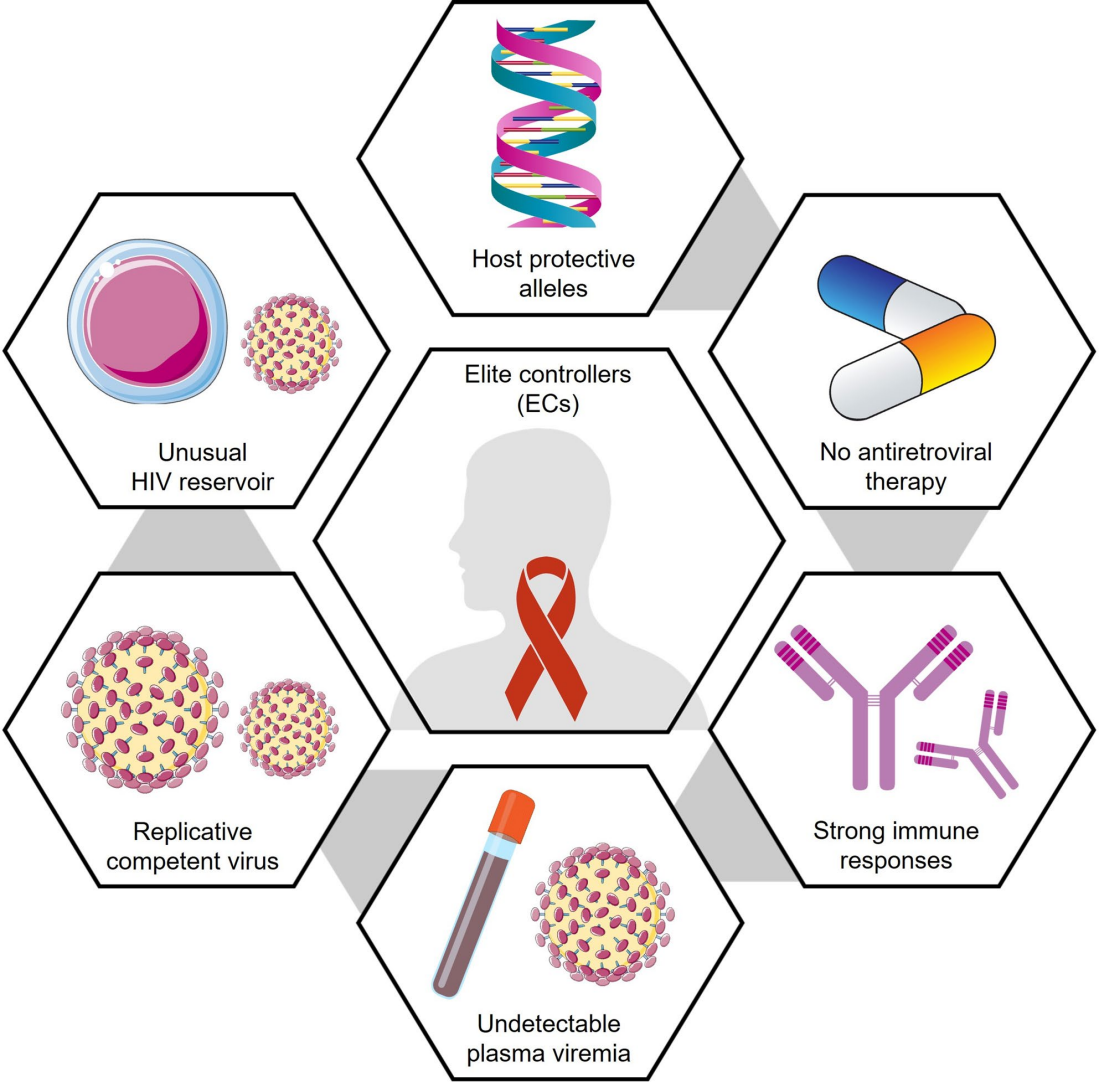
Key determinants of elite control of HIV infection. Schematic representation of the multifactorial biology underlying spontaneous viral suppression in ECs. ECs maintain undetectable plasma viremia and stable CD4 + T cell counts in the absence of ART. Contributing factors include the presence of host protective alleles, a small and transcriptionally restricted HIV reservoir, replication-competent but immune-contained virus, and potent innate and adaptive immune responses. Together, these features establish durable control of HIV replication without ART and provide a model for functional cure strategies

A consistent pattern emerges across regions: low reservoir burden, effective immune surveillance, and restricted viral diversity underpin durable control. Two US patients maintained suppression for 12 and 24 years with only transient “blips” (56 and 93 copies/mL), suggesting stable immune containment of transcriptional activity [85]. Comparable cases in India further highlight population diversity. Some individuals maintained asymptomatic infection for decades, with stable CD4 + T cell counts [86], while others exhibited partial control before experiencing immunologic decline [87]. Collectively, these reports demonstrate that the ability to control HIV without ART arises from multiple biological routes, including genetic, immunologic, and virologic, reflecting convergent evolution toward functional remission, as illustrated in Fig. 2 [88, 89]. Representative EC and EEC cases across cohorts and regions are summarized in Table 2.

**Table 2:** Summary of key reported cases and cohorts of elite controllers (ECs) and exceptional elite controllers (EECs), integrating both population-level and individual-level evidence

Cohort/case	Region/population	Key features	Mechanistic insights	Duration of control	References
International HIV Controllers Study	Multinational (Europe, Americas, Africa; *n* ≈ 1,500)	Defined global frequency (< 1% PLHIV); identified protective alleles (HLA-B*57:01, B*27:05, B*81:01)	Genetic determinants of control linked to optimized peptide presentation via HLA class I	Cross-sectional (median > 7 years)	Pereyra et al., 2010
ANRS CO21 CODEX Cohort	France; *n* > 300 ECs	Characterized long-term ECs; ~30% lost virological control; elevated IL-10 and IFN-γ profiles	Identified immune activation patterns predicting loss of control	Median 15 years (follow-up)	Lambotte et al., 2005
Spanish Exceptional Elite Controller (EEC) Cohort	Spain; *n* = 20 EECs	Documented > 25 years of ART-free remission; >90% defective proviral genomes; absence of replication-competent virus	Polyfunctional CD8 + T cell and NK cell responses; protective HLA-B57, B58, B27	> 25 years	Casado et al., 2020
Sydney Blood Bank Cohort (SBBC)	Australia; transfusion recipients	Infection with *nef/3’LTR*-deleted HIV-1; sustained aviremia > 35 years	Viral attenuation, HLA-B57, and strong CTL responses contribute to near-sterilizing control	> 37 years	Zaunders et al., 2019
African Female EC1 & EC2	South Africa; female ECs	Two women maintained undetectable viremia for > 7 years without ART	EC1: strong CD8 + T cell responses; EC2: pre-existing CD4 + T immunity; multiple protective HLAs (B*57:03, B*81:01, B*44:03)	> 7 years	Moosa et al., 2018
U.S. Long-term EC Cases	United States; 2 individuals	Sustained viral suppression 12 and 24 years with minor “blips” (56–93 copies/mL)	Strong CTL memory and low reservoir activity	12 to 24 years	Jiang et al., 2020
Indian EC Cases	North and South India	Asymptomatic infection for > 20 yrs; stable CD4 + counts; no ART	Possible genetic and immunologic resilience; limited reservoir seeding	6 to 21 years	Sankaranantham, 2022; Shanmukhappa et al., 2020
San Francisco Patient (Loreen Willenberg)	USA; female EEC	No intact provirus in > 1.5 billion PBMCs; ART-naïve since 1992	Natural sterilizing cure; robust polyfunctional CD8 + T responses	> 28 years	Mendoza et al., 2012
Esperanza Patient	Argentina; female EEC	Undetectable viremia > 8 years; no replication-competent virus in > 1.1 billion PBMCs and placental cells	Near-sterilizing cure; immune containment with restricted provirus landscape	> 8 years	Turk et al., 2022

Among all reported cases, two stand out as the most complete examples of natural HIV control: the San Francisco and Esperanza patients. Both demonstrated complete suppression of replication-competent virus without the need for stem cell transplantation or sustained ART [90]. The San Francisco patient, Loreen Willenberg, was announced in 2020 as the “world’s first known person to naturally beat HIV” [91, 92]. She tested positive for HIV in 1992 but never experienced symptoms of acute retroviral syndrome, despite known risk exposures, including instances of unprotected intercourse [82]. Her medical history included basal cell carcinoma and nephrolithiasis, but she remained otherwise healthy. Over the years, her HIV serological tests were intermittently indeterminate or weakly positive. Remarkably, she remained ART-naïve, with CD4 + T cell counts consistently above 1,200 cells/µL and plasma viral loads persistently undetectable. Deep sequencing analyses of more than 1.5 billion peripheral blood mononuclear cells (PBMCs) failed to identify intact proviral sequences, supporting the possibility of a sterilizing HIV cure [93].

A second case was reported in Argentina: the Esperanza patient, diagnosed with HIV-1 in March 2013 [94]. Throughout eight years of follow-up, she exhibited no clinical or laboratory evidence of HIV-1–related disease, with plasma viral loads consistently below the detection threshold. ART was administered for six months during her pregnancy (September 2019 to March 2020) but discontinued postpartum, after which viral loads remained undetectable. Extensive sequencing of 1.188 billion PBMCs and 503 million placental mononuclear cells failed to detect intact proviral genomes. Only seven defective proviruses, some derived from clonally expanded cells, were identified. Viral outgrowth assays using 150 million resting CD4 + T cells yielded no replication-competent virus, and no HIV-1 RNA was detected in 4.5 ml of plasma.

These landmark cases confirm that sterilizing cure is biologically possible, albeit exceedingly rare. Features distinguishing EECs, such as the San Francisco and Esperanza patients, include [55]: (i) undetectable plasma viremia and preserved CD4 + T cell counts, (ii) viral determinants and co-receptor tropism, (iii) restricted viral persistence, including very low levels of cell-associated HIV DNA/RNA and absence of replication-competent virus, (iv) host immunogenetic factors, and (v) potent HIV-specific T cell and cytokine responses. A comparative summary of these two cases is presented in Table 3, emphasizing shared biological features that define the most complete examples of natural control.

**Table 3:** Comparative clinical and virological characteristics of the San Francisco and Esperanza patients

San Francisco patient	Criteria	Esperanza patient
• Both are female patients • Both presented with weak/incomplete antibody responses • Both presented with high CD4 + T cell count • Both expressed wild-type CCR5 • Both presented with low frequency of HIV-specific CD8 + T cells		
71 years old (as of 2025)	Age	35 years old (as of 2025)
1992 (38 years old)	Year of diagnosis	2013 (23 years old)
ART was not prescribed	ART regimen	ART was prescribed for a 6-month period from September 2019 to March 2020 due to the pregnancy
One detectable viral load (93 copies/plasma ml) within a total of 39 tests performed (as of Jan 2020)	Viral load	No detectable viral load, within 14 tests (as of April 2022)
HLA-B*57:01	Protective alleles	No protective allele
Less than 1 copy/ml	Ultrasensitive plasma RNA	Less than 0.2 copy/ml
Undetectable	qPCR cell-associated (CA) DNA and/or RNA PBMCs	Undetectable
Undetectable	qPCR CA DNA and/or RNA gut-associated lymphoid tissue (GALT)	Not done
Undetectable (HIC: 238 million CD4 + T cells, qVOA: 340 million CD4 + T cells)	Replication-competent virus assay (high-input quantitative coculture, HIC or quantitative viral outgrowth assay qVOA)	Undetectable (qVOA: 150 million CD4 + T cells)
No intact provirus/few defective provirus (14 million resting CD4 + T cells)	Intact Proviral DNA Assay (IPDA)	Not done
No intact provirus/19 defective provirus (1.5 billion PBMCs)	Full-Length Individual Proviral Sequencing (FLIP-seq) PBMC	No intact provirus/7 defective provirus (1.18 billion PBMCs)
No intact or defective provirus (7.72 million cells from the GALT)	FLIP-seq Tissue	No intact or defective provirus (503 million cells from the placenta)

A related phenomenon known as post-treatment control (PTC) illustrates that durable suppression can also be achieved following structured therapy, defined as planned, closely monitored initiation and interruption of ART under clinical supervision [95–97]. Unlike spontaneous controllers, PTCs achieve remission after therapeutic intervention, most often when ART is initiated during acute or early infection, limiting reservoir seeding and preserving immune integrity. PTCs maintain undetectable or low-level viremia for years after ART interruption, despite the presence of intact proviruses. Mechanistically, ECs and PTCs share several overlapping features: small and transcriptionally silent viral reservoirs, preserved immune surveillance, and reduced systemic inflammation. However, PTCs typically lack the protective HLA alleles enriched among ECs, suggesting that remission in PTCs depends more on treatment timing and immune preservation than on genetic predisposition. Together, these observations highlight complementary biological routes to ART-free remission, one naturally established and the other therapy-induced, providing critical insights for designing functional cure strategies. Understanding the convergences and divergences among ECs, EECs, and PTCs provides a crucial framework for cure research, as summarized in Table 4.

**Table 4:** Comparison of the defining features, Immunovirological correlates, and translational significance of elite controllers (ECs), exceptional elite controllers (EECs), and post-treatment controllers (PTCs)

Feature	Elite controllers (ECs)	Exceptional elite controllers (EECs)	Post-treatment controllers (PTCs)
Definition	Spontaneous suppression of plasma HIV RNA to < 50 copies/mL without ART.	Subset of ECs with ultra-low/undetectable viremia by ultrasensitive assays and absent or near-absent replication-competent virus.	Durable suppression of viremia for months–years after structured ART interruption.
Approx. frequency	< 1% of PLHIV.	Extremely rare (< < 1%).	~ 5–10% among those treated very early (cohort-dependent).
Typical ART exposure	ART-naïve (control without therapy).	ART-naïve or minimal/irrelevant exposure.	Prior ART; usually initiated during acute/early infection and interrupted.
HLA/genetic correlates	Enrichment of protective HLA class I alleles (HLA-B*57, B27, B*58), some CCR5Δ32 heterozygosity.	Similar or stronger enrichment of protective alleles in many cases.	Typically lack protective HLA enrichment.
Immune responses	Highly polyfunctional, high-avidity CD8 + T cells; NK-cell involvement; preserved CD4 + T cell help.	Very strong polyfunctional CD8 + T cell and NK responses; robust antibody-dependent effector functions.	Partial restoration of CD8 + memory and preserved immune function post-ART; variable breadth/avidity.
Viral reservoir	Small, low-diversity reservoir; replication-competent virus often detectable at very low frequency.	Ultra-low or absent inducible replication-competent reservoir; high proportion (> 90%) defective proviruses.	Reduced reservoir size due to early ART, but intact proviruses persist.
Viremia (plasma)	Undetectable by standard assays (< 50 copies/mL); occasional blips.	Undetectable even by single-copy assays (< 1 copy/mL) in many cases.	Undetectable or low-level viremia after ART interruption (variable durability).
Durability of control	Often lifelong in some, but ~ 25–30% may lose control long-term (cohort-dependent).	Typically extremely durable (> 25 years in reported cases).	Durable in a minority; depends on the timing of ART and host factors.
Clinical relevance/translational insight	Natural model of host-mediated remission, which informs immune-based therapies (vaccines, checkpoint modulation).	Proof-of-principle that a near-sterilizing cure is biologically possible, which informs reservoir-elimination strategies.	Demonstrates that early ART can enable durable remission, which supports combined early-treatment and immune-boost approaches.

## Mechanisms of elite control: viral, intrinsic, genetic, and immune factors

The mechanisms underlying durable, treatment-free HIV suppression in ECs are multifactorial, reflecting a delicate equilibrium between host defenses and viral adaptability. Increasing evidence suggests that elite control arises not from a single mechanism, but from the interplay of viral, intrinsic, genetic, and immune determinants that together sustain long-term remission [98–101].

### Viral reservoir characteristics and dynamics viral control

Early hypotheses proposed that ECs and LTNPs harboured replication-defective or attenuated viruses, particularly those with deletions in *nef* or *env* genes, which limited viral fitness [102, 103]. However, many ECs carry replication-competent HIV-1 without such defects [104, 105]. Ultra-sensitive assays have even detected low-level plasma viremia, sometimes exceeding that seen in ART-suppressed individuals [106, 107]. These observations suggest that viral attenuation alone cannot account for control, and that the host immune environment plays a significant role in limiting replication.

The viral reservoir in ECs is characteristically small, genetically homogeneous, and transcriptionally silent. Proviruses tend to integrate into repressive chromatin regions, limiting reactivation potential [19]. This restricted reservoir contrasts sharply with that of typical progressors, in whom proviruses occupy transcriptionally active loci. EECs often harbour more than 90% defective proviruses with minimal replication-competent virus, suggesting near-complete reservoir silencing. Collectively, these data suggest that elite control reflects both active immune pressure and passive integration into genomic “safe zones” that together maintain viral latency.

### Immune function and effector mechanisms of viral control

The immune architecture of ECs is distinguished by robust yet balanced antiviral responses. CD4 + T cell counts are typically preserved [108, 109], although a gradual decline can occur due to thymic exhaustion or persistent activation [110–112]. Dendritic cells exhibit enhanced antigen presentation but restrained proinflammatory cytokine release, partly regulated by unique leukocyte immunoglobulin-like receptor (LILR) expression patterns [113, 114]. ECs demonstrate highly polyfunctional HIV-specific CD8 + T cells capable of simultaneous degranulation and secretion of perforin, granzyme B, interferon gamma (IFN-γ), tumor necrosis factor alpha (TNF-α), interleukin 2 (IL-2), and macrophage inflammatory protein-1β (MIP-1β) [113, 115–117]. The quality of these cytotoxic T-cell responses, defined by multifunctionality and rapid cytolytic capacity, correlates more closely with control than cell number. Emerging evidence also highlights the role of natural killer (NK) cells, which cooperate with T cells through killer-cell immunoglobulin-like receptor (KIR)-HLA interactions and mediate antibody-dependent cellular cytotoxicity (ADCC), thereby reinforcing innate-adaptive strategies [118–120].

Humoral immunity adds another layer of control. Although ECs generally show lower neutralizing antibody titres than viraemic individuals, their B cell compartment maintains remarkable breadth and potency. Broadly neutralizing antibodies (bNAbs) such as LN01, isolated from EC germinal-centre B cells, recognize conserved Env epitopes across clades [121–124]. Robust T follicular helper (Tfh) cell activity preserves memory B-cell function, facilitating durable antibody production and enhancing ADCC responses. Together, these effector mechanisms create a finely tuned immune equilibrium that suppresses viral replication without causing excessive inflammation.

### Protective alleles and host genetic determinants of viral control

Genetic factors strongly influence the likelihood of achieving elite control. Genome-wide association studies (GWAS) have identified single-nucleotide polymorphisms associated with spontaneous viral suppression, including rs2395029 in HCP5 (linked to HLA-B57:01), rs9264942 upstream of HLA-C, and rs3869068 in ZNRD1/RNF39 [125–128]. Among these, HCP5 exerts the strongest effect through linkage disequilibrium with the HLA-B57:01 allele [129, 130]. Protective HLA class I alleles, such as HLA-B27 and HLA-B57, facilitate superior peptide presentation to cytotoxic T lymphocytes (CTLs), promoting the recognition of conserved Gag epitopes where escape mutations impose significant fitness costs [131–134]. Conversely, alleles such as HLA-B35 and B53 are associated with a more rapid progression. Beyond HLA, interactions between HLA-Bw4 motifs and KIRs on NK cells enhance the clearance of infected lymphocytes [135]. Importantly, not all individuals carrying protective alleles achieve control, and some without them remain long-term non-progressors, indicating that immunogenetics set the stage for, but do not solely determine, viral containment [136].

### Host intrinsic restriction factors and mechanisms of viral control

ECs exhibit elevated expression of several intrinsic antiviral restriction factors that limit HIV replication independently of adaptive immunity [137]. ECs demonstrate increased expression of several restriction genes, including apolipoprotein B mRNA editing enzyme, catalytic polypeptides like 3 (APOBEC3), Ctr9, Paf1/RNA polymerase II complex component (CTR9), tripartite motif family (TRIM), and Schlafen 11 (SLFN11), the latter being strongly upregulated in CD4 + T cells of ECs compared with ART-suppressed or non-controller individuals [138]. SLFN11 interferes with HIV replication in a codon usage–specific manner [139–144], and disrupts DNA damage response pathways by depleting type II transfer RNAs, thereby inhibiting viral protein synthesis [145, 146]. It also impairs Gag–plasma membrane interactions, reducing viral assembly [147].

Cyclin-dependent kinase inhibitor 1 A (CDKN1A/p21) restricts HIV by suppressing enzymes involved in deoxyribonucleotide triphosphate (dNTP) biosynthesis, thereby limiting reverse transcription [148, 149]. CDKN1A modulates HIV-1 replication through multiple pathways, including regulation of the dNTP pool and inhibition of host CDK2, which supports reverse transcription [150, 151]. The increase in CDKN1A expression leads to the downregulation of several enzymes (RNR2, TYMS, and TK1) involved in dNTP biosynthesis, which decreases the dNTP pool size required for reverse transcription, thereby restricting HIV-1 replication [152, 153]. CDKN1A also enhances the activity of SAMHD1, which decreases intracellular dNTP pools and prevents HIV DNA synthesis [154–162]. These restriction mechanisms impose early, cell-intrinsic barriers that reduce viral replication before the adaptive immune response is activated.

**Fig. 3:**
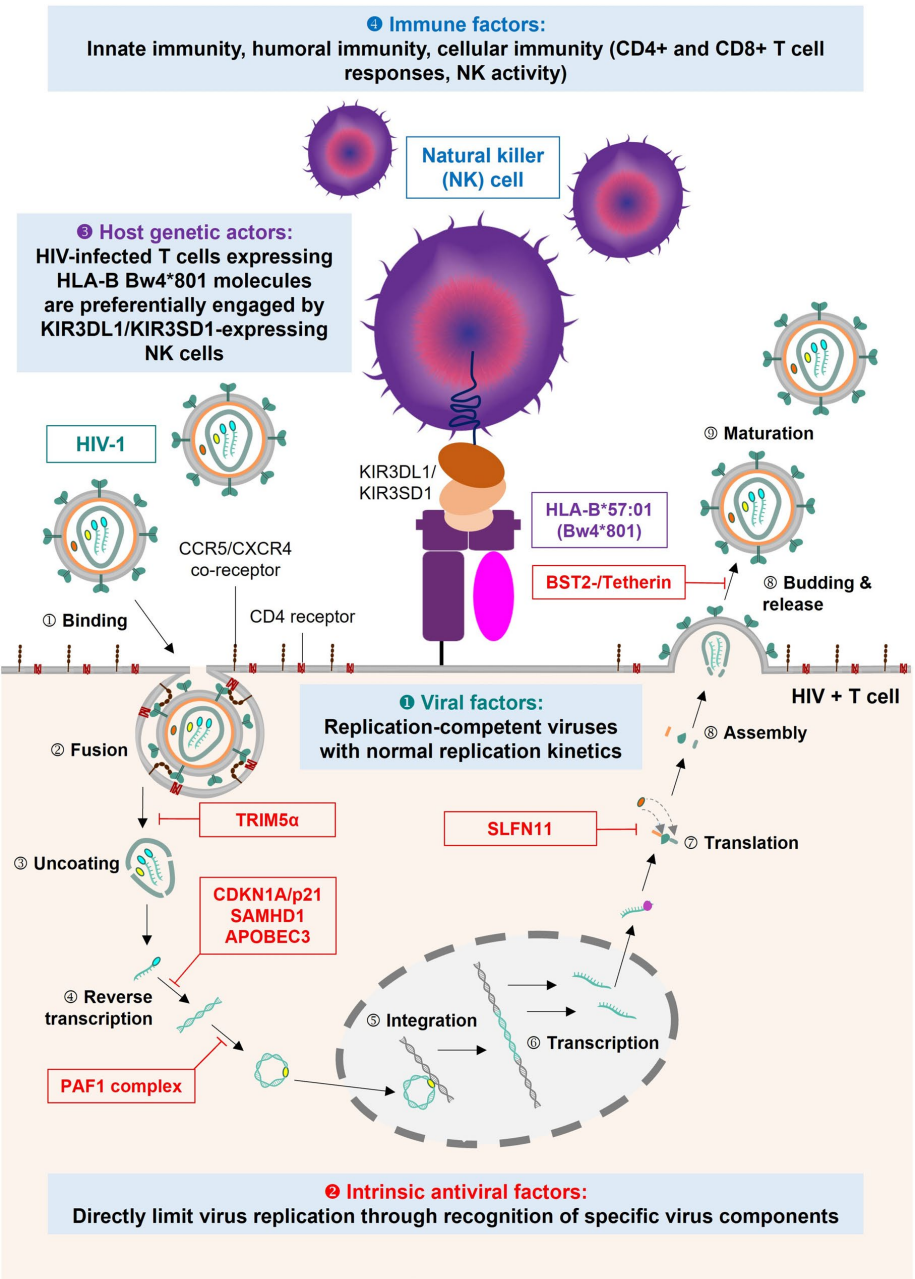
Mechanisms of host-mediated viral suppression in ECs. Schematic illustration of the integrated mechanisms contributing to spontaneous HIV control in elite controllers. (1) Viral factors: replication-competent viruses with normal replication kinetics are effectively contained through host restriction pathways. (2) Intrinsic antiviral factors: cellular proteins, including TRIM5α, APOBEC3, SAMHD1, CDKN1A/p21, SLFN11, BST2/Tetherin, and the PAF1 complex, interfere with multiple stages of the HIV life cycle, from reverse transcription to viral assembly. (3) Host genetic factors: protective alleles such as HLA-B*57:01 (Bw4801) and interactions with KIR3DL1/KIR3DS1-expressing NK cells enhance immune recognition of infected targets. (4) Immune factors: coordinated innate, cellular (CD4 + and CD8 + T cell), and humoral responses sustain long-term viral suppression and immune homeostasis. Collectively, these mechanisms establish a durable equilibrium between host immunity and viral persistence, defining the biological foundation of elite control

Taken together, these findings confirm that elite control results from the convergence of viral, genetic, immune, and intrinsic factors that jointly maintain a state of functional remission. Limited reservoir size, protective HLA-driven immunity, and strong restriction factor activity form a multilayered defense network that balances viral persistence and immune regulation. Rather than a static endpoint, elite control represents a dynamic equilibrium shaped by continuous coadaptation between the host and virus. This integrated framework provides a natural blueprint for translational research. Therapeutic strategies that combine early ART initiation (to minimize reservoir formation), induction of restriction factors, and enhancement of elite-like immune responses, particularly polyfunctional CD8 + T cells, NK cell synergy, and ADCC, may reproduce the spontaneous remission observed in ECs. A conceptual overview of these interacting mechanisms is presented in Fig. 3, illustrating how viral, host, and immune determinants align to maintain durable HIV suppression.

## Future directions: translating elite control into functional cure strategies

More than four decades after the first reports of HIV/AIDS, ART continues to transform the lives of PLHIV. Although ART has drastically improved survival and quality of life, lifelong treatment remains limited by adherence challenges, drug-related toxicities, and the persistence of latent viral reservoirs. Consequently, achieving a definitive cure remains the ultimate goal for global HIV eradication. In contrast to the rapid progress seen in vaccine development for emerging infections such as SARS-CoV-2, the quest for an effective HIV vaccine has proven far more complex, with multiple large-scale trials ending without efficacy [163–165]. These setbacks underscore the need for alternative approaches, particularly those aimed at achieving a functional cure, defined as durable, ART-free remission of infection [89, 166].

ECs provide compelling proof of concept that such remission is biologically possible. By sustaining undetectable replication without therapy, ECs illustrate how host genetics, intrinsic restriction mechanisms, and potent cellular and humoral immunity can collectively maintain viral suppression. Insights from ECs reveal key determinants of durable control, including protective HLA alleles and KIR-HLA interactions, the expression of restriction factors such as SLFN11, CDKN1A/p21, and SAMHD1, and highly polyfunctional CD8 + T cell and NK cell responses that prevent viral rebound [167]. These mechanisms define the correlates of protection and offer tangible targets for therapeutic replication. Advances in cell- and gene-based approaches, including gene editing, immune modulation, and therapeutic vaccination, offer promising opportunities to replicate the protective mechanisms observed in ECs.

Gene-editing approaches, such as CCR5 disruption or proviral excision using CRISPR/Cas systems, emulate the natural resistance seen in ECs carrying the CCR5Δ32 mutation or restricting viral integration to transcriptionally silent genomic regions [168–170]. Therapeutic vaccines and immune-checkpoint modulators seek to re-establish the polyfunctional, high-avidity CD8 + T cell and NK cell responses characteristic of ECs [171–173], while bNAbs mirror the potent antibody-dependent cellular cytotoxicity that contributes to elite viral suppression [174–176]. Latency-reversing or “block-and-lock” agents target the reservoir dynamics observed in ECs, where proviruses are deeply silenced or integrated into repressive chromatin [177, 178]. Together, these interventions aim to recreate the integrated immune equilibrium achieved naturally by ECs, thereby reducing the reservoir size, enhancing immune surveillance, and maintaining durable remission without the need for continuous ART.

The study of ECs thus provides a biological roadmap for cure research. By decoding both the durability and the fragility of natural control, future interventions can reinforce protective immune networks while minimizing chronic inflammation and immune exhaustion. EC research highlights the principle that durable remission arises from the synergistic action between genetic resilience, immune precision, and viral containment, rather than relying on monotherapy. Future work must focus on identifying biomarkers of sustained remission, validating EC-inspired therapies across diverse populations and HIV subtypes, and translating mechanistic insights into scalable, globally accessible interventions. Integrating genetic and immune correlates into clinical trial design will be essential to accelerate progress toward safe and sustainable functional cures. In the spirit of World AIDS Day, which calls for reflection, remembrance, and renewed commitment, EC research stands as a testament to how scientific discovery can transform a rare immunological phenomenon into a universal therapeutic paradigm. As the global HIV community continues to strive toward eradication, lessons from ECs remain central to transforming rare biological phenomena into achievable therapeutic outcomes.

## Data Availability

No datasets were generated or analysed during the current study.
